# Operative and antibiotic treatment of cervical spondylodiscitis: a retrospective bicentric outcome analysis

**DOI:** 10.1007/s00701-026-06929-w

**Published:** 2026-06-03

**Authors:** Kavi Raj Chataut, Christoph Schwartz, Matthias Matejka, Jens Lehmberg, Arwin Rezai, Alexander Romagna

**Affiliations:** 1 Department of Neurosurgery, München Klinik Bogenhausen, Munich, Germany; 2 Department of Neurosurgery, University Hospital Salzburg, Paracelsus Medical University Salzburg, Salzburg, Austria; 3 Department of Neurosurgery, University Hospital Innsbruck, Medical University of Innsbruck, Medizinzentrum Anichstrasse 35, 6020 Innsbruck, Austria

**Keywords:** Cervical spondylodiscitis, Morbidity, Mortality, Outcome, Surgery

## Abstract

**Objective:**

Cervical spondylodiscitis is a rare but complex disease due to its potential for severe neurological impairments and post-infectious deformity. The reported mortality rate ranges from 5 to 10%. Radical surgical treatment is usually followed by antibiotic therapy. There is, however, no uniform treatment guideline. Implant choice, extent of fusion and duration of treatment vary across centers. The aim of this study is to analyze a decade of treatment of patients suffering from primary cervical spondylodiscitis to gain insights for improving existing treatment strategies.

**Methods:**

This is a retrospective analysis of surgically treated patients suffering from cervical spondylodiscitis between January 2014 and December 2024 in two academic spine centers. Following ethical approval, pre- and postoperative imaging and clinical course were evaluated. Outcome analysis included assessment of implant complications, corrections of sagittal and coronal profiles, complication rates, length of hospital stay, neurological deficits, pain intensity, and 1-year survival. Clinical and radiological reassessments were performed at or shortly after completion of antibiotic therapy.

**Results:**

The study included 33 patients (52% males) with a median age at diagnosis of 68 years (IQR 59–76 years). The most common pathogen was staphylococcus aureus (39%). All patients underwent surgical debridement, predominantly by ventral decompression and fusion (90%). Following surgery, a significant number of patients showed an improvement in cervical radiculopathy and neck pain (*n* = 24/33, *p* < 0.0001). One patient required revision surgery due to screw misplacement during the same hospital stay. The average inpatient stay was 23 days (21 days), with 15 patients requiring postoperative treatment in the intensive care unit. Within a median clinical follow-up of three months there was radiological evidence of implant-loosening in five (15%) patients. Three (9%) patients died within one year because of sepsis, all of whom were older than 75 years and had significant comorbidities (ASA score > 3).

**Conclusion:**

Our study supports the effectiveness of surgical treatment for cervical spondylodiscitis in short to medium-term outcome analysis, particularly through ventral decompression and fusion. The significant improvement in radiculopathy and pain reduction post-surgery underscores the treatment benefits. However, 1-year mortality is still high in geriatric patients with relevant comorbidities. Future studies should focus on long-term outcomes and complications to further optimize the treatment strategy for this dangerous condition.

**Supplementary Information:**

The online version contains supplementary material available at 10.1007/s00701-026-06929-w.

## Introduction

Cervical spine spondylodiscitis is a rare but complex neurosurgical disease [6, 10, 18]. The incidence of cervical spine spondylodiscitis has been increasing and is reported up to 5.8 per 100,000 per years in some studies [20]. Despite being less prevalent than thoracic and lumbar spondylodiscitis, it is associated with an increased rate of post-infectious deformity, secondary neurological deficits, and substantially higher rates of associated morbidity [5, 10, 14, 15, 20, 22]. Additionally, the disease is still associated with a significant mortality rate reported of up to 5–10% in some case series [15].

The common treatment consists of radical surgery with instrumentation of the affected segment followed by antibiotics [20]. However, implant choice, extent of fusion and duration of treatment vary across centers and there is no uniform treatment guideline. Also, there is a lack of studies on the outcome of surgical management in terms of pain reduction, improved neurologic function and improved stability of the vertebral column. This study aims to evaluate ten years of treatment outcomes in patients with primary cervical spondylodiscitis in order to identify opportunities to improve current treatment strategies. In particular, we assessed neurological and radiological outcomes following surgical intervention. Additionally, we analyzed how outcomes differed according to risk factors, comorbidities and patient age groups.

## Methods

### Patients and clinical assessment

In this retrospective study, we analyzed data of surgically treated patients suffering from cervical spondylodiscitis between January 2014 and December 2024 in two distinct academic centers. This study was performed in line with the principles of the Declaration of Helsinki. After approval was granted by the Ethics Committee of the LMU University of Munich (No. 22–0585) and the PMU University of Salzburg (PMU-EK-2025–0031), we evaluated the clinical course as well as pre- and postoperative imaging. We assessed patient demographics and characteristics at the time of presentation, which included age, sex, body mass index (BMI), comorbidities including alcohol consumption and smoking, previous operations, history of radiation as well as chemotherapy. Several objectifiable assessment tools were applied for patient characterization. Comorbidities and patients’ functional status were objectified using the frailty index known as the Modified 5 Item Frailty Index (mFI-5) described elsewhere [16]. Perioperative risks were assessed by the American Society of Anesthesiologists (ASA) Score [7]. Additionally, the Charlson Comorbidity Index (CCI) was used to potentially predict mortality in patients with multiple comorbidities [3]. Neurological impairment was objectively assessed using the Frankel grade [4]. Clinical and radiological short-term outcomes were assessed at or shortly after completion of antibiotic therapy. No study-specific treatment, surgical procedures and/or imaging was performed. Prior informed consent to participate was obtained from all patients and no funding was provided to support this work.

### Surgery

The primary goals of surgical treatment are thorough debridement of infected tissue, stabilization of the spine, and decompression of neural structures [20]. The most commonly used approach is an anterior cervical discectomy and fusion (ACDF) with or without cervical plating. In cases with evidence of vertebral body destruction, a corpectomy, is performed followed by an implantation of a cylindrical expandable cage supported by additional plating. Another alternative is posterior spinal instrumentation, which is also generally complemented to anterior spinal instrumentation for added stability [2].

The surgical indication, choice of approach, extent of resection, and implant selection were determined through internal case discussions within the two participating neurosurgical departments. In this study, we evaluated surgery-related parameters including surgical approach, implants used, operative duration, anesthesia time, and surgery-associated complications.

### Imaging

We analyzed preoperative, immediate postoperative and follow-up imaging, including magnetic resonance imaging (MRI) and computed tomography (CT)/X-ray, to evaluate implant-related complications and changes in sagittal alignment in all patients. Sagittal alignment was assessed by measuring the local sagittal angle (defined as the angle between the superior endplate and inferior endplate of the affected vertebrae) and the C2–C7 angle (defined as the angle between the inferior endplate of the C2 vertebral body and the inferior endplate of the C7 vertebral body) [9].

### Outcomes

The primary outcome of this study was neurological improvement following surgery. Secondary outcomes included; i) radiological outcome and implant-complications, ii) microbiological as well as histological assessments, iii) length of hospital stay (including duration of intensive care unit treatment) and length of antibiotics therapy (intravenous as well as oral), and iv) one year mortality (retrieved from hospital database).

### Statistics

Along with descriptive analysis of patient characteristics an outcome analysis was performed, which included assessment of implant complications, corrections of sagittal and coronal profiles, complication rates, length of hospital stay, neurological deficits, pain intensity and one-year mortality.

Statistical analyses were conducted by using GraphPad Prism 8. The parameters were compared by using unpaired *t*-tests (for normally distributed data), Fisher’s Exact Test for Categorical variables, Mann–Whitney test (for not normally distributed data), and Chi-Square test (for categorical data). A *p*-value of < 0.05 was considered statistically significant.

## Results

### Patient characteristics and surgical details

Demographic data are presented in Tables 1 and 2. We included 33 patients (17 males) in our analyses and the median age at surgery was 68 years (IQR 59–76 years). The most common symptoms were nuchalgia (*n* = 28) and radiculopathy (*n* = 25); eight (24%) patients showed signs of cervical myelopathy. Additionally, 21 patients were diagnosed with bacteremia on admission. None had undergone prior surgery of the cervical spine and/or radiotherapy. Assessment of lifestyle-related patient characteristics showed that most patients considered themselves nonsmokers (*n* = 27) and did not report an excessive alcohol consumption (*n* = 27).

**Table 1:** Basic patient characteristics

Basic patient characteristics Total patients *n* = 33	
Median Age in years	
(IQR)	68 (59–76)
Sex	
Male	17 (52%)
Female	16 (48%)
Median ASA Score	3
(IQR)	(3 −4)
Median Charlson- Comorbidity Index—CCI	4
(IQR)	(2 −6)
Affected segments	
C1- C2	0
C2- C3	1
C3- C4	5
C4- C5	8
C5- C6	15
C6- C7	7

**Table 2:** Patients' preoperative status

ID	Center	Age	Sex (0 = male 1 = female)	Comorbidities*	mFi −5 Index	ASA	CCI	Alcohol consumption (1 = yes/0 = no)	Smoking (1 = yes/0 = no)	Chemotherapy (1 = yes/0 = no)	Affected number of cervical segments	Epidural Abscess (1 = yes/0 = no)	Radiculopathy (1 = yes/0 = no)	Myelopathy (1 = yes/0 = no)	Nuchalgia (1 = yes/0 = no)	Frankel Grade
1	2	76	1	1, 4, 5	2	4	6	0	0	0	1	1	1	0	1	C
2	2	64	1	0	0	2	3	0	0	0	1	0	1	0	1	E
3	2	57	1	0	0	3	2	0	0	0	1	1	1	0	1	C
4	2	66	1	8	0	2	2	0	0	0	3	0	1	1	1	D
5	2	63	0	4, 13, 8	3	4	6	1	1	0	3	1	1	1	1	C
6	2	58	1	4, 8, 10	2	4	4	0	0	0	3	1	0	0	1	B
7	2	73	1	4, 8, 11	1	3	4	0	0	0	2	0	1	0	1	D
8	2	39	0	0	0	2	0	0	0	0	1	1	1	0	1	D
9	2	39	1	8	0	4	0	0	1	0	1	1	1	1	1	C
10	2	68	1	4, 8	1	4	2	0	0	0	2	1	1	1	1	A
11	1	66	1	1	1	2	3	0	0	0	2	1	1	1	1	E
12	1	69	1	2, 4, 5	2	3	5	0	0	0	2	1	1	0	1	E
13	1	68	1	3	1	2	2	0	0	0	3	1	0	0	1	E
14	1	59	0	4, 13	3	3	4	0	0	0	2	1	1	1	1	A
15	1	73	1	1, 2	3	2	11	0	0	1	2	0	1	0	1	E
16	1	58	1	4, 13	3	3	2	0	0	0	2	1	0	0	1	D
17	1	81	1	2, 4	2	3	7	0	0	1	2	1	1	0	1	E
18	1	47	0	11	1	3	3	0	0	1	2	1	1	0	1	D
19	1	60	0	0	1	3	4	0	0	0	3	1	1	0	1	A
20	1	58	0	4	1	3	2	0	0	0	1	1	1	0	1	E
21	1	67	0	4, 13	3	3	3	1	1	0	1	0	1	0	0	D
22	1	53	0	5, 9	1	3	4	1	0	0	4	1	1	0	0	E
23	1	76	0	2, 4,5, 10	2	3	8	0	0	0	2	1	1	0	1	D
24	1	70	0	4	2	4	5	0	1	0	2	0	0	0	0	E
25	1	86	0	1, 4, 5, 10	3	4	7	0	0	0	1	1	1	0	1	D
26	1	80	1	4	2	3	5	1	0	0	2	1	1	0	1	C
27	1	77	1	4	2	4	7	0	0	1	1	1	0	1	0	C
28	1	83	0	4	2	4	9	0	0	0	1	0	1	0	1	B
29	1	67	0	0	1	4	2	0	0	0	2	1	1	0	1	E
30	1	78	0	2	1	2	5	0	0	0	1	0	0	0	0	B
31	1	68	0	2	1	2	3	0	0	0	3	1	0	0	1	C
32	1	74	0	0	1	2	3	0	0	0	3	1	1	1	1	E
33	1	82	0	1, 2, 4	3	3	8	1	1	0	3	0	0	0	1	E

*Comorbidities:

1 = diabetes, 2 = malignancy, 3 = rheumatic disease/sondyloarthropathy, 4 = arterial hypertension/heart failure/HRS, 5 = renal insufficiency, 6 = autoimmune disease, 7 = osteoporosis, 8 = substance abuse, 9 = liver cirrhosis, 10 = stroke, 11 = neurodegenerative disease, 12 = gastrointestinal disease, 13 = lung disease

The assessed patient cohort had a preoperative median mFI-5 of 1(IQR 1–2) with no patient exceeding a score of 3. The median preoperative ASA score was 3 (IQR: 2.5–3); six patients were ASA II, twelve were ASA III, and five were ASA IV. The recorded median CCI was 4 (IQR: 3–7; Table 1).

In the majority of patients (*n* = 24; 73%), either one or two cervical segments were affected (1-level: *n* = 11; 2-level: *n* = 13, 3-level: *n* = 8, 4-level: *n* = 1). The most frequently affected segment was C5/6 (Table 1). Multilocal spondylodiscitis was seen in 7/33 (21%) of patients with lumbosacral being the most common additional site. Preoperative MRI, which was used for the diagnostics, showed evidence of additional epidural abscess in 24 (73%) patients. Thirteen (39%) patients underwent whole spine MRI showing discitis in additional segments in 7/13 of these cases. Predominantly patients with symptoms and neurological deficits suggesting distant spinal infection underwent whole spine MRI. The affected segments included the lumbosacral (*n* = 4), lumbar only (*n* = 1), and thoracolumbar (*n* = 2) regions. All patients underwent surgery on the affected spinal segments. Two patients had abscess evacuation alone, while the remaining patients received additional dorsal instrumentation of the involved segments. The sequence of surgeries was determined based on the predominance of neurological deficits: specifically. Three patients were operated before the cervical procedure and four patients underwent surgery afterward.

In none of the evaluated cases, a preoperative percutaneous biopsy was performed. All patients underwent radical surgical debridement, predominantly by ventral decompression and fusion (88%). Three patients underwent combined ventral and dorsal fusion, whereas one patient was treated with dorsal instrumentation only. Surgical times differed based on number of segments and approach used. The mean operation time was of 171± 75 min (1-level: 145 ± 99 min; 2-level: 183 ± 46 min; 3-level: *n* = 206 ± 29 min; 4-level: 140 min) (Table 3) and average anesthesia time was 231 ± 83 min (1-level: 223 ± 102 min; 2-level: 246 ± 51 min; 3-Level: 297 ± 67 min; 4-level: 234 min). In all patients a surgical drain was placed. There were no intraoperative complications. The surgical approach and implants used are included in Table 4.

**Table 3:** Approach and levels

Surgery	
**Approach**	**Number of Patients**
Anterior	29
Dorsal	1
Combined	2
**Number of cervical levels operated**	**Average Operation time in minute ± SD (number of patients)**
Total	171 ± 75 (*n* = 33)
1-Level	145 ± 99 (*n* = 11)
2-Level	183 ± 46 (*n* = 13)
3-Level	206 ± 29 (*n* = 8)
4-Level	140 (*n* = 1)

**Table 4:** Surgical details

ID	Center	Surgical approach (1 = ventral, 2 = dorsal, 3 = ventrodorsal)	Operation	Implants used*	Plate **
1	2	1	Vertebral body replacement of C5 and C6	Iliac crest bone graft	Pina plate
2	2	1	ACDF C4/5	Iliac crest bone graft	HISS plate
3	2	1	ACDF C6/7	no implant used	no plate used
4	2	1	Vertebral body replacement of C5 and C6	Pina Cage	HISS plate
5	2	1	Vertebral body replacement of C5 and C6	Pina Cage	HISS plate
6	2	1	Vertebral body replacement of C4, C5 and C7	Pina Cage	HISS plate
7	2	1	Vertebral body replacement of C6	Pina Cage	Trinica plate
8	2	1	ACDF C4/5 and C5/6	NEOCIFA Cage	HISS plate
9	2	2	Laminectomy from C3 to Th1	no implant used	no plate used
10	2	1	Vertrebal body replacement of C5 and C6	Pina Cage	Skyline plate
11	1	1	Vertrebal body replacement of C5 and C6	ADD Cage	Trinica plate
12	1	1	Vertrebal body replacement of C5 and C6	ADD Cage	Trinica plate
13	1	1	Vertrebal body replacement of C5 and C6	ADD Cage	Trinica plate
14	1	1	Vertrebal body replacement of C4 and C5	ADD Cage	Trinica plate
15	1	3	Vertrebal body replacement of C5 and C6 + posterior rod-screw fixation	ADD Cage + **SYNAPSE**	Trinica plate
16	1	1	Vertrebal body replacement of C6 with partial corpectomy C7	ADD Cage	Trinica plate
17	1	1	Vertrebal body replacement of C5 and C6	ADD Cage	Trinica plate
18	1	1	Vertrebal body replacement of C3 and C4	ADD Cage	Reflex plate
19	1	3	Vertebral body replacement of C5 + posterior rod-screw fixation from C4 to Th 1	ADD Cage + Neon^3^	Trinica plate
20	1	1	ACDF 5/6	SynCage	no plate used
21	1	1	ACDF C6/7	ACIS Cage	Trinica plate
22	1	1	ACDF C3/4, C4/5, C5/6, and C6/7	ACIS Cage	Signus plate
23	1	1	ACDF C6/7 and C7/Th1	ACIS Cage	Trinica plate
24	1	1	ACDF C5/6 and C6/7	ACIS Cage	no plate used
25	1	1	ACDF C6/7	ACIS Cage	Trinica plate
26	1	1	ACDF C4/5 and C5/6	ACIS Cage	Trinica plate
27	1	1	ACDF C6/7	SynCage	Trinica plate
28	1	1	ACDF C4/5	ACIS Cage	Trinica plate
29	1	1	ACDF C4/5 and C5/6	SynCage	no plate used
30	1	1	ACDF C5/6	ACIS Cage	no plate used
31	1	1	ACDF C3/4, C4/5 and C5/6	ACIS Cage	Trinica plate
32	1	1	Vertebral body replacement at C4 with cage at C4/5 and C5/6 with plate	ADD Cage + ACIS Cage	Trinica plate
33	1	1	ACDF C5/6 and C6/7	ACIS Cage	Trinica plate

* ACIS cage (DePuy Synthes, Raynham, MA, USA; material: PEEK); ADD cage (Ulrich Medical, Ulm, Germany; material: titanium alloy); Biomet NeoCif cage (Zimmer Biomet, Warsaw, IN, USA; material: PEEK); Neon^3^ posterior rod-screw fixation system (Ulrich Medical, Ulm, Germany; material: titanium alloy); PINA cage (Ulrich Medical, Ulm, Germany; material: titanium alloy); SynCage (DePuy Synthes, Raynham, MA, USA; material: titanium); SYNAPSE™ posterior rod-screw fixation system (DePuy Synthes Spine, Raynham, MA, USA; material: titanium alloy)

** Pina plate (Zimmer, Minneapolis, MN, USA; material: titanium alloy); Trinica plate (Zimmer Spine, Minneapolis, MN, USA; material: titanium alloy); SIGNUS plate (SIGNUS Medizintechnik, Alzenau, Germany; material: titanium alloy); Reflex plate (Stryker Spine, Kalamazoo, MI, USA; material: titanium alloy); HISS plate (Hofer GmbH & Co KG, Fürstenfeld, Austria; material: titanium alloy); Skyline plate (Medtronic, Minneapolis, MN, USA; material: titanium alloy)

### Clinical and radiological outcomes

**Fig. 1:**
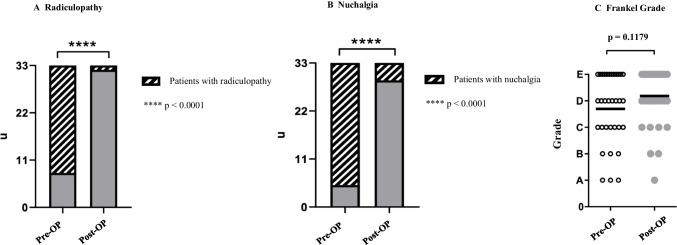
Neurological outcomes

**Fig. 2:**
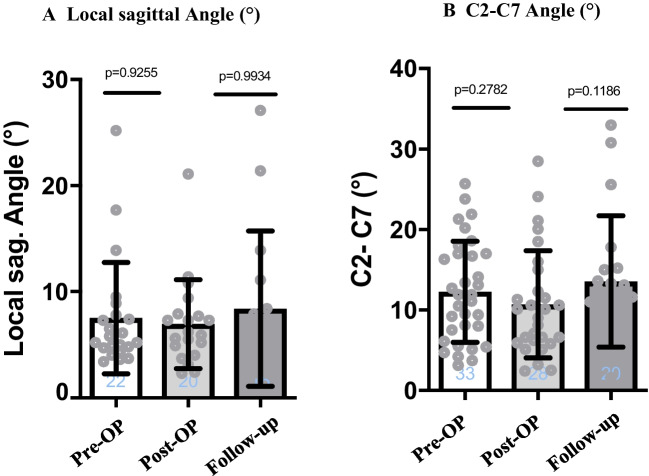
Radiological outcomes

**Fig. 3:**
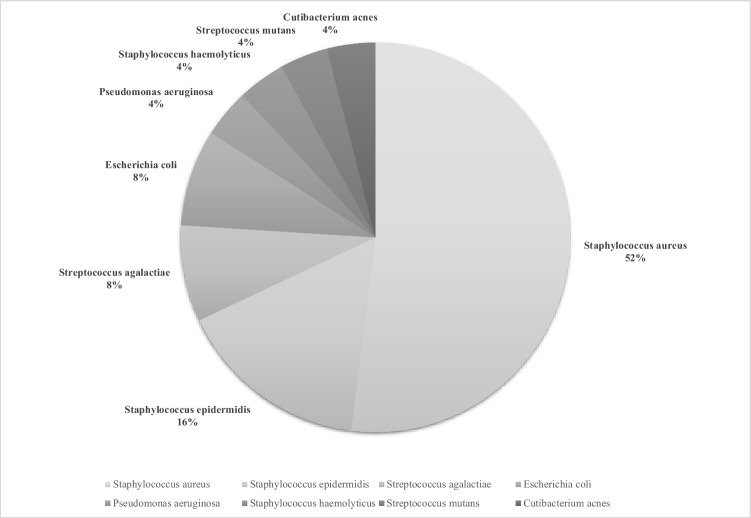
Pathogen

**Fig. 4:**
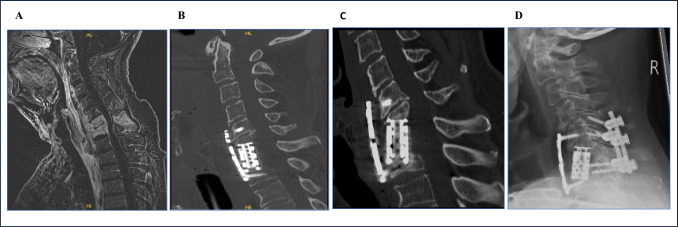
Screw loosening and cage subsidence

Immediately postoperatively, a significant number of patients showed an improvement in cervical radiculopathy and neck pain (*p* < 0.0001). The overall neurological status, objectified by the Frankel grade, did not show a significant difference between pre- and post-operative (*p* = 0.4286) with most patients already showing good preoperative neurological function (Frankel grade E or D; Fig. 1). After surgery, both the local sagittal angle (pre op: 7.5° ± 5.2°, post op: 7.0° ± 4.2°; *p* = 0.9255) and the C2-C7 angle (pre op: 12.3° ± 6.3°, post op: 10,7° ± 6.7°; *p* = 0.2782) showed no significant differences compared to the preoperative condition (Fig. 2). Postoperative CT scans showed ventral screw misplacement in one patient. Median follow-up time was 3 months (IQR 2–4 months). A trend toward improvement in the sagittal alignment/angle could be seen, while analyzing the local sagittal angle (post op: 7.0° ± 4.2° vs. follow-up: 8.4° ± 7.3°; *p* = 0.9934) and the C2-C7 angle (post op: 10.7° ± 6.7° vs. follow- up: 13,7° ± 8.2°; *p* = 0.1186) without reaching statistical significance (Fig. 2). Furthermore, there was radiological evidence of implant-loosening in five cases (15%) during follow-up (Fig. 3). Three cases presented with only screw loosening, and two cases involved cage subsidence with screw loosening (Table 5, Fig. 4). Apart from one case with residual numbness, paresis, and difficulty with fine motor skills and one case of paresis, all patients presented with nuchalgia. Univariate analysis using Fisher’s exact test did not demonstrate statistically significant associations between implant loosening and epidural abscess (*p* = 0.29), sepsis (*p* > 0.99), alcohol use (*p* > 0.99), or smoking (*p* > 0.99). Although all cases of implant loosening occurred in patients with an epidural abscess, this factor was also common among patients without loosening, limiting its predictive value.

**Table 5:** Screw loosening and cage subsidence

	Implant Loosening	Age	Sex	Epidural abscess (1 = yes/0 = no)	Sepsis (1 = yes/0 = no)	Alcohol consumption (1 = yes/0 = no)	Smoking (1 = yes/0 = no)	mFi	CCI	ASA	Initial surgery	Cage used**	Plate used***	Symptoms	Revision surgery
1	screw loosening	58	f	1	1	0	0	0	0	4	Vertebral body replacement of C4- C7	Pina cage	HISS plate	Nuchalgia	4 months
2	cage subsidence and screw loosening	66	f	1	0	0	0	1	3	2	Vertebral body replacement of C5 and C6	ADD cage	Trinica plate	Nuchalgia	3 months
3	cage subsidence and screw loosening	58	f	1	0	0	0	3	2	3	Vertebral body replacement of C6 with partial corpectomy C7	ADD cage	Trinica plate	Nuchalgia, Numbness, Paresis	4 months
4	cage subsidence and screw loosening	60	m	1	0	0	0	1	4	3	Vertebral body replacement of C5	ADD cage	Trinica plate	Nuchalgia, Paresis	1 month
5	screw loosening	76	m	1	1	0	0	2	8	3	ACDF C6/7 and C7/Th1	ACIS cage	Trinica plate	Nuchalgia	8 months
	p-value *	0.18		0.29	> 0.99	> 0.99	> 0.99	0.75	> 0.99	0.25					

*Fisher’s Exact Test for Categorical variables (Epidural abscess, Sepsis, Alcohol, Smoking) and Mann–Whitney U test (non-parametric) for Continuous variables (mFI, CCI, ASA)

**ADD cage (Ulrich Medical, Ulm, Germany; material: titanium alloy);

ACIS cage (DePuy Synthes, Raynham, MA, USA; material: PEEK)

*** Trinica plate (Zimmer Spine, Minneapolis, MN, USA; material: titanium alloy)

### Microbiological findings, hospital course and mortality

In all cases, intraoperative swabs were sent for microbiological and histopathological assessments. Pathogens could be identified in 24 (73%) samples. Among patients in whom no pathogen was found were two cases that underwent prior antibiotic treatment. The most commonly identified pathogens were Staphylococcus aureus (39%) and Staphylococcus epidermidis (12%; Fig. 3). Histological assessment showed signs of infection in 18 (55%) patients. Detailed reporting of pathogens (in microbiological specimens and blood cultures), antibiotic regimens (both intravenous and oral) and inflammatory parameter (C-reactive Protein; CRP pre op and after antibiotic therapy) are to be found in supplement table I.

The average hospital stay was 23 days, with 15 patients requiring postoperative treatment in the intensive care unit (average stay four days). Patients received intravenous antibiotics for a mean duration of 31 days and subsequent oral antibiotics for a mean duration of 62 days (Table 6). In total, four (12%) patients died within one year after surgery; of those one died due to an unrelated intracranial hemorrhage, and three (9%) patients died of sepsis/multiorgan failure (Table 7). All of these patients were older than 75 years and suffered from significant comorbidities (i.e., ASA score > 3, CCI > 7).

**Table 6:** Hospital stay and antibiotic treatment

Length of Hospital stay and antibiotic therapy	
Hospital Stay	Average days ± SD (number of Patients)
Total	23 ± 21 (*n* = 33)
ICU	4 ± 10 (*n* = 15)
Antibiotics	Average days ± SD
Intravenous	31 ± 26
Oral	62 ± 48

**Table 7:** Mortality

Age	Sex	Comorbidities/History of	mFi −5 Index	ASA	CCI	Blood culture (preoperativ)	Affected segments	Epidural abscess	Died after (days)	Died of
86	male	Diabetes, arterial hypertension, heart failure, stroke, history of hip & knee arthroplasty	3	4	7	positive	1	yes	9	Sepsis > Multi-organ Failure
77	female	Multiple myeloma	0	4	7	positive	1	yes	45	Sepsis > Multi-organ Failure
76	male	Prostatic cancer, arterial hypertension, heart failure, Stroke	2	3	8	positive	2	yes	263	Sepsis > Multi-organ Failure

## Discussion

A drastic increase of pyogenic (cervical) spondylodiscitis has been noted over the last years [8, 17, 20]. This subsequently led to an increased clinical debate regarding optimal treatment [5, 20]. Our study addressed this issue by evaluating outcome measures in patients treated surgically at two academic spine centers. Our principal results demonstrated: 1) improvement in clinical symptoms (such as neck pain and radiculopathy), 2) favorable changes in radiological parameters and 3) increased mortality in geriatric patients with multiple comorbidities.

Our study supports the effectiveness of radical surgical treatment for cervical spondylodiscitis. Clinical improvement regarding nuchalgia and radiculopathy was seen especially in cases undergoing ventral decompression and fusion combined with subsequent long-term antibiotic therapy. This is in line with earlier similar studies [4, 9, 14]. A metanalysis including 21 studies and data from 10,954 patients supports our results and emphasizes the necessity of risk reduction of relapse/failure, mortality and length of hospital stay through early surgery [19]. However, no significant improvement was observed in the Frankel grade in contrary to that of similar study from Gao and colleagues, where significant improvements in the Frankel grading were observed in patients with neurological deficits during follow-up [4]. This may be explained by a relatively low preoperative Frankel index of patients in our study. Of note, the decision for surgical treatment of patients with relatively mild neurological signs and symptoms was not based solely on preoperative Frankel index, but also on factors such as epidural abscess formation, progressive infection, pain progression, mechanical instability, extent of bony destruction, and the need for adequate debridement and tissue diagnosis. This rationale for surgical treatment depicts practical clinical setting.

Radiologically, we observed favorable trends in cervical alignment, measured by local sagittal angle and C2–C7 angle [13]. While statistical significance was not reached, this may be explained by the small sample size and the absence of severe preoperative malalignment. Similar findings were reported by Tsai et al. and Kim et al., who demonstrated improved kyphotic deformity and segmental alignment following early surgical treatment [9, 21]. Pseudarthrosis rates in our cohort were low. However, 15% of patients required revision surgery for implant loosening, all of whom had epidural abscesses, indicating a more complicated disease course [11]. This suggests that while an epidural abscess may be associated with implant loosening, it is not sufficient on its own to reliably predict implant loosening. As clinical and radiological reassessment were performed at or shortly after completion of antibiotics therapy, these findings underscore the importance of monitoring long-term outcomes in this population.

One-year mortality was notably higher in elderly patients with multiple comorbidities and elevated ASA and CCI scores. Previous studies reported comparable mortality rates, but failed to stratify patients by preoperative risk [14, 15]. Although we do not find any association between mortality and the modified frailty index, in contrast to Alas et al., we found a clear association of mortality with ASA and CCI scores [1]. Motov et al. discussed comparable results in their study, highlighting that higher ASA and CCI scores were related to poor clinical outcome [11].

The surgical complication rate in our entire study group was low. This might reflect both advances in surgical and intensive care management and the relatively good baseline health of our cohort. Nevertheless, hospital stays were prolonged, with nearly 45% of patients requiring postoperative intensive care, emphasizing the complexity of managing this condition even in patients with minimal frailty.

Regarding our microbiological results, our study showed Staphylococcus aureus as the most common causative pathogen which most frequently affected the C5/6 segment. These findings are consistent with prior studies [12, 14, 20].

Notably, our analysis provides important insights but it is subject to some limitations: a relatively small number of patients limits the statistical power of the study and may reduce the generalizability of the findings. Still, cervical spondylodiscitis is a rare disease, which given the steady rise of the incidence, is very likely to increase. Another limitation is the retrospective design of our study as well as standardization of surgical techniques and diagnostic follow-up. However, this heterogeneity might depict real clinical scenarios and enhance the general applicability of the results.

It would be valuable to compare this cohort with conservatively treated patients as comparative outcome analysis, which was also limited because of the design of the study. Furthermore, future prospective studies using standardized radiographic protocols should include comprehensive sagittal parameters such as T1 slope and sagittal vertical axis too, as they provide a more comprehensive view of cervical sagittal balance and may influence long-term clinical and mechanical outcomes. Since this assessment was constrained by the retrospective study design of our study.

Although the follow-up period in our study captures the clinically relevant time point at which treatment response is typically evaluated, it remains relatively short for a complex condition such as spondylodiscitis, in which late recurrence or delayed instability may occur. Besides that, our study lacks systematic diagnostic evaluation of infection pathways and inflammatory parameters.

A multicenter study with standardized protocols, extended follow-up period and a larger patient population could help address these limitations.

## Conclusion

Our study supports the effectiveness of radical surgical treatment for cervical spondylodiscitis. One-year mortality is high in geriatric patients with relevant comorbidities. Given the demographic change and increasing incidence of cervical spondylodiscitis, our data reinforce the need to consider age, as well as ASA and CCI scores, when planning surgery. Prospective studies with standardized diagnostic and treatment protocols are needed to assess long-term outcomes and complications to further optimize the treatment strategy for this dangerous condition.

## Supplementary Information

Below is the link to the electronic supplementary material.

ESM 1 Supplementary Material 1 (DOCX 25.9 KB)

ESM 2 Supplementary Material 2 (DOCX 27.3 KB)

## Data Availability

All data that support the findings of this study are available from the corresponding author upon reasonable request.

## Code availability

A code availability is not applicable.
