# Supplementary material for: Operative and antibiotic treatment of cervical spondylodiscitis: a retrospective bicentric outcome analysis
Source: Acta Neurochir (Wien). 2026 Jun 3;168(1):207. doi: 10.1007/s00701-026-06929-w (PMC13447335; doi:10.1007/s00701-026-06929-w)
Supplement: Supplementary file 1 [file 701_2026_6929_MOESM1_ESM.docx]

| **ID** | **Center** | **Pathogen in microbiological specimen**  **(0= no pathogen detected)** | **Pathogen in Blood culture**  **(0= no pathogen detected)** | **Antibiotics (i.v.)** | **Antibiotics (p.o.)** | **CRP pre-OP (mg/l)** | **Duration of Antibiotics (days)** | **CRP at last follow up (mg/l)** |
| --- | --- | --- | --- | --- | --- | --- | --- | --- |
| 1 | 2 | Staphylococcus aureus | 0 | Lidaprim | No oral Antibiotics | 459 |  | N/A (28.9 at discharge) |
| 2 | 2 | 0 | 0 | Fosfomycin, Roceohin | Clindamycin | 40.8 | 19 | 14 |
| 3 | 2 | Staphylococcus aureus | 0 | Cefuroxim, Rocephin | Targocid, Fucidinsäure | 209.2 | 11 | N/A (81.4 at discharge) |
| 4 | 2 | Staphylococcus aureus | 0 | Vancomycin, Ciprofloxacin | N/A | 8.6 |  | N/A (31 at discharge) |
| 5 | 2 | 0 | 0 | Ampicillin/Sulbactam | Ampicillin/Sulbactam | 47.8 | 56 | 0.9 |
| 6 | 2 | Staphylococcus aureus and Streptococcus agalactiae | Staphylococcus aureus and Streptococcus agalactiae | N/A | N/A | 314 | N/A | 10.8 |
| 7 | 2 | 0 | 0 | Unasyn, Fosfomycin | Rifoldin | 41.4 | 13 | 27.4 |
| 8 | 2 | Staphylococcus aureus | 0 | Ceftriaxon, Rocephin, Metronidazol | Floxapen | 410 | 71 | 4 |
| 9 | 2 | Staphylococcus aureus | Staphylococcus aureus | Cefuroxin, Fosfomycin and Cefalexin | Floxapen | 16.8 | 64 | 0.1 |
| 10 | 2 | Staphylococcus aureus | Staphylococcus aureus | Cefuroxim, Fosfomycin and Cefazolin | Rifoldin, Eusaprim | 440 | 72 | 3 |
| 11 | 1 | 0 | 0 | Clindamycin | Clindamycin | 13 | 187 | N/A |
| 12 | 1 | 0 | 0 | Clindamycin | Clindamycin | 7.3 | 135 | 6.4 |
| 13 | 1 | 0 | 0 | Clindamycin | Clindamycin | 91 | 42 | NA (26.5 at discharge) |
| 14 | 1 | Pseudomonas aeroginosa | 0 | Meropenem | Ciprofolxacin | 27.6 | 112 | NA (3.3 at dscharge) |
| 15 | 1 | Staphylococcus epidermidis | 0 | Vancomycin, Ceftriaxon, Rifampicin | No oral Antibiotics | 40.3 | 84 | 3.5 |
| 16 | 1 | Staphylococcus aureus | 0 | Clindamycin | Clindamycin | 66.4 | 96 | 16.7 |
| 17 | 1 | 0 | 0 | Piperacillin/tazobactam and Clindamycin | Clindamycin | 16 | 74 | 0.7 |
| 18 | 1 | 0 | Staphylococcus aureus | Staphylex | Rifampicin | 37 | 51 | 18 |
| 19 | 1 | Staphylococcus haemolyticus | Staphylococcus haemolyticus | Meropenem and Clindamycin | Clindamycin | 90.8 | 33 | 4.9 |
| 20 | 1 | Streptococcus mutans | 0 | Clindamycin and Ceftriaxon | Clindamycin | 155.5 | 208 | 5.1 |
| 21 | 1 | Staphylococcus aureus | 0 | Meropenem+ Rifampicin > Cefazolin+ Rifampicin | Flucloxacillin and Rifampicin | 136 | 63 | NA (5.8 at discharge) |
| 22 | 1 | 0 | Streptococcus agalactiae | Ceftriaxon | Ampicillin, Cefuroxim | 107 | 104 | NA (78 at discharge) |
| 23 | 1 | Staphylococcus aureus | Staphylococcus aureus | Piperacillin/tazobactam > Linezolid and Clindamycin | Clindamycin | 111 | 120 | 20.6 |
| 24 | 1 | Staphylococcus aureus | Staphylococcus aureus | Cefazolin, Clindamycin | Levofloxacillin | 29.9 | 53 | NA (1.1 at discharge) |
| 25 | 1 | Staphylococcus epidermidis | 0 | Clindamycin, Rifampicin, Gentamycin | No oral Antibiotics | 137.7 | 9 | 134.2 |
| 26 | 1 | Escherichia coli | Escherichia coli | Meropenem, Ceftriaxon | Ciprofolxacin | 309.1 | 122 | 2.9 |
| 27 | 1 | Staphylococcus aureus | Staphylococcus aureus | Flucloxacillin, Fosfomycin | No oral Antibiotics | 190.8 | 51 | 49.5 |
| 28 | 1 | 0 | Staphylococcus aureus | Clindymycin | Rifampicin | 82 |  | 7.7 |
| 29 | 1 | Streptococcus agalactiae | 0 | Clindamycin > Ceftriaxon | Amoxicillin, Rifampicin | 154.8 | 106 | NA (27.6 at discharge) |
| 30 | 1 | Staphylococcus epidermidis | 0 | Fosfomycin, Cefazolin | Cotrimaxozol, Rifampicin | 19.7 | 84 | NA (10.3 at discharge) |
| 31 | 1 | Escherichia coli | 0 | Ampicillin/Sulbactam | Ciprofolxacin | 79.1 | 90 | 0.5 |
| 32 | 1 | Staphylococcus aureus | Staphylococcus aureus | Flucloxacillin> Daptomycin | Levofloxacillin, Rifampicin | 231.3 | 44 | NA (25.4 at discharge) |
| 33 | 1 | Staphylococcus epidermidis and Cutibacterium acnes | 0 | Piperacillin/tazobactam | Levofloxacillin, Rifampicin | 46.5 | 84 | NA (238.8 at discharge) |

N/A = not available
