# Supplementary material for: Operative and antibiotic treatment of cervical spondylodiscitis: a retrospective bicentric outcome analysis
Source: Acta Neurochir (Wien). 2026 Jun 3;168(1):207. doi: 10.1007/s00701-026-06929-w (PMC13447335; doi:10.1007/s00701-026-06929-w)
Supplement: Supplementary file 2 [file 701_2026_6929_MOESM2_ESM.docx]

**Supplement II:**

a) Modified 5 Item Frailty Index (mFI-5) is a prognostic tool based on five comorbidities (i.e., hypertension, heart failure, chronic obstructive pulmonary disease (COPD), diabetes, and non-independent functional status). [8]

| **Risk factors** | **Point** |
| --- | --- |
| Congestive heart failure in the 30 days before surgery | 1 |
| History of severe chronic obstructive pulmonary disease or pneumonia | 1 |
| Hypertension requiring medication | 1 |
| Diabetes mellitus with oral agents or insulin | 1 |
| Non-independent functional health status at the time of surgery | 1 |
|  |  |
| Total | 5 |

| **Class** | **Definition** |
| --- | --- |
| **ASA I** | A normal healthy patient |
| **ASA II** | Mild systemic disease |
| **ASA III** | Severe systemic disease |
| **ASA IV** | Constant threat to life |
| **ASA V** | Moribund; not expected to survive |
| **ASA VI** | Declared brain-dead |
| **"E"** | Emergency |

b) American Society of Anesthesiologists (ASA) Score classifies severity of coexisting diseases and the functional status of the patient prior surgery. [9]

| **Point** | **Condition** |
| --- | --- |
| 1 Point | Myocardial Infarction, Congestive Heart Failure, Peripheral Vascular Disease, Cerebrovascular Disease (including TIA), Dementia, Chronic Pulmonary Disease, Connective Tissue/Rheumatic Disease, Peptic Ulcer Disease, Mild Liver Disease, Diabetes (without end-organ damage). |
| 2 Points | Hemiplegia or Paraplegia, Moderate/Severe Renal Disease, Diabetes with end-organ damage (e.g., retinopathy, neuropathy), Any Tumor (localized), Leukemia, Lymphoma. |
| 3 Points | Moderate or Severe Liver Disease (e.g., cirrhosis with portal hypertension). |
| 6 Points | Metastatic Solid Tumor, AIDS (HIV infection with complications). |

c) Charlson Comorbidity Index (CCI) is aa index which predicts mortality in patients with multiple comorbidities. This Index ranges from 1 to 6 points, in which one point is given to for each medical condition, such as heart disease, diabetes, kidney disease, and cancer. [10]
d) Frankel grade objectifies neurological impairments, in which grading goes from A to E: Grade A for the patients with complete motor and sensory paralysis and Grade E for the patients with no neurological deficits. [11]

| **Frankel Grading** | **Description** |
| --- | --- |
| **A** | Complete motor and sensory loss |
| **B** | Complete motor loss, incomplete sensory loss |
| **C** | Incomplete motor loss without practical use |
| **D** | Incomplete motor loss, able to ambulate with or without walking aids |
| **E** | Free of neurological symptoms |
